# Supplementary material for: Neural crest induction requires SALL4-mediated BAF recruitment to lineage specific enhancers
Source: Development. 2025 Dec 19;152(24):dev205248. doi: 10.1242/dev.205248 (PMC12752504; doi:10.1242/dev.205248)
Supplement: Supplementary information [file develop-152-205248-s1.pdf]

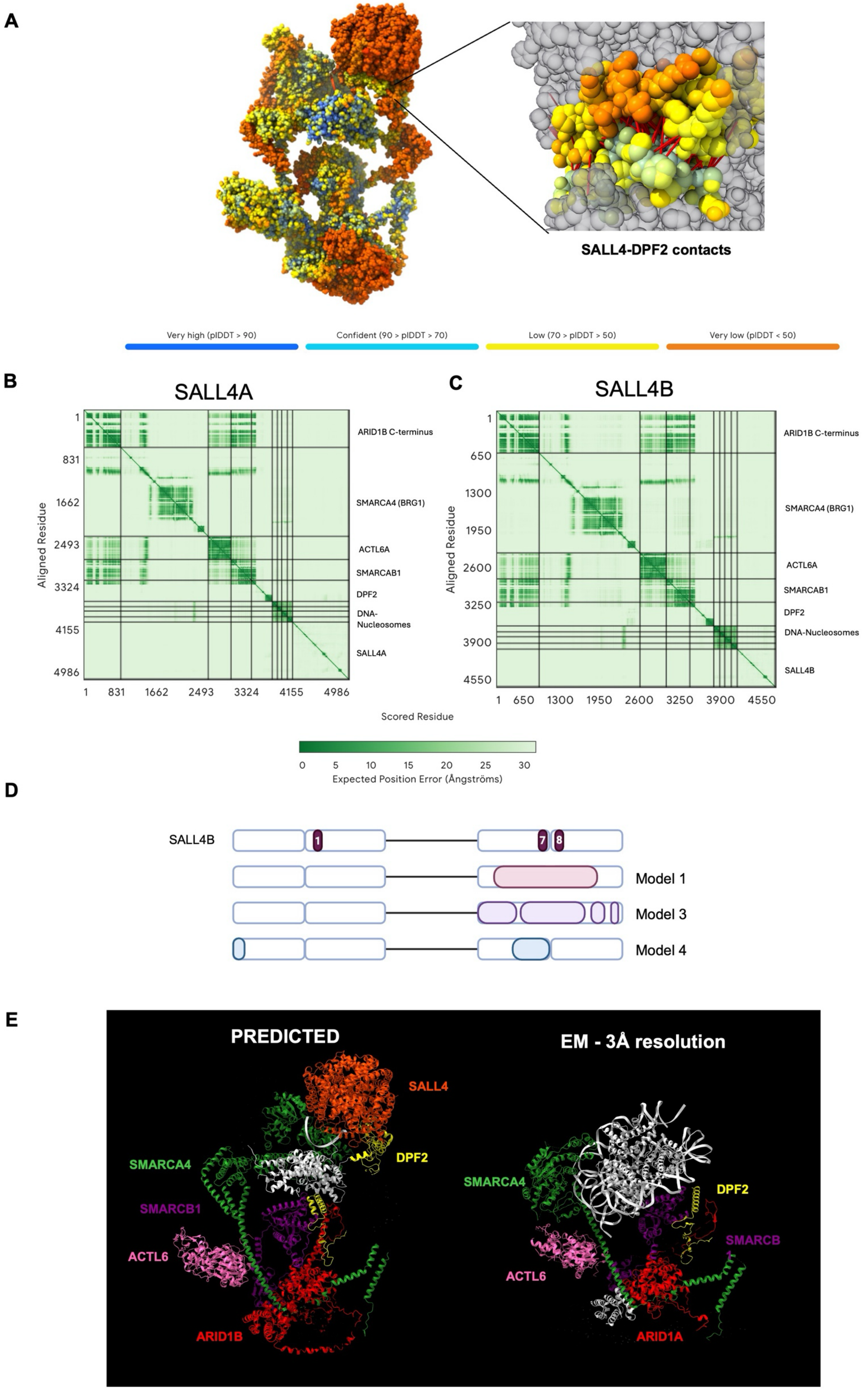

Development • Supplementary information

Fig. S1. SALL4BB is not predicted to interact with BAF *in silico*

(A) (Left) Visual representation of the confidence score pLDDT (predicted Local Distance Difference Test) for each residue of the AlphaFold-predicted complex, with color gradients indicating prediction confidence (blue: very high confidence, yellow: low confidence, orange/red: very low confidence). (Right) Close-up view of the SALL4A-DPF2 interface highlighting key interacting residues in red.

(B) AlphaFold predicted aligned error (PAE) plot for model 0 of SALL4A. The plot shows the expected positional error in Ångströms for each residue pair.

(C) AlphaFold predicted aligned error (PAE) plot for model 0 of SALL4B. The plot shows the expected positional error in Ångströms for each residue pair.

(D) SALL4B protein sequences. C2H2 zinc fingers annotations are highlighted in dark purple and numbered. Visual representation of output models of SALL4B (models 1, 3, and 4). Interactions between SALL4B and DPF2 are highlighted in pink, light purple, and light blue. Made with BioRender.com.

(E) Comparison between the predicted BAF-SALL4A in silico structure and an experimentally identified structure of the human BAF Base module (PDB: 6LTH).

Development • Supplementary information

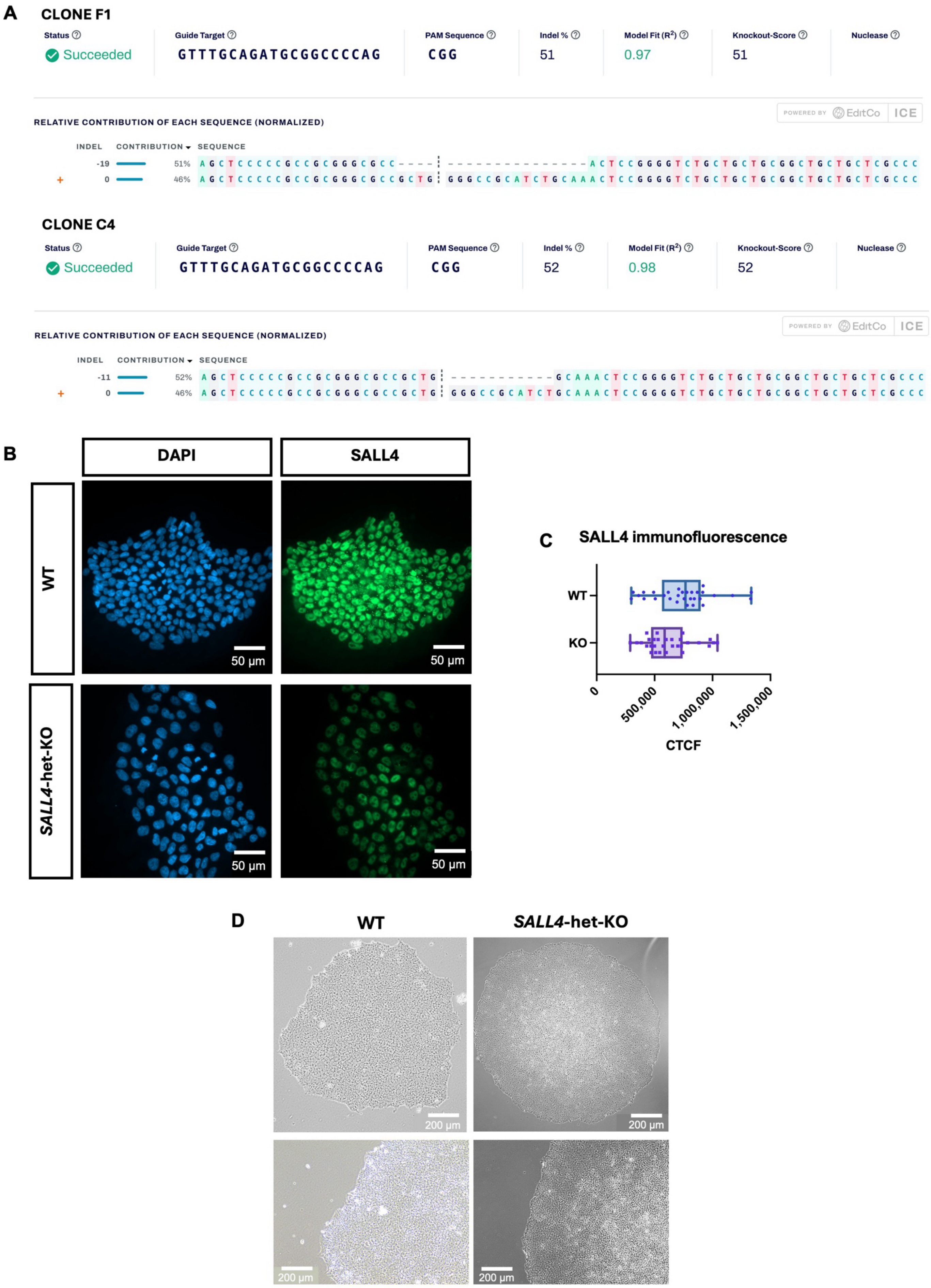

Development • Supplementary information

Fig. S2. SALL4 does not bind to at-rich regions when relocating to CNCC-associated enhancers

(A) Sanger sequencing reports of the two iPSC *SALL4*-het-KO clones used in this study: clone F1 (19-bp deletion, top) and clone C4 (11-bp deletion, bottom). The data were analyzed using the ICE (Inference of CRISPR Edits) tool to quantify CRISPR-Cas9 editing efficiency. Editing resulted in an overall indel frequency of 51% and 52%, respectively. The sequence traces show the relative contribution of each allele, normalized across the population, with deleted bases indicated by dashed lines. Cut sites are represented by black vertical dotted lines, and the wild-type sequence is marked by a "+" symbol on the far left.

(B) Immunofluorescence labeling quantifying the expression of SALL4 in WT and clone F1 iPSC lines. Scale bar: 50 μm.

(C) Box plot showing Corrected Total Cell Fluorescence (CTCF) values quantifying and comparing SALL4 expression in WT and SALL4-het-KO (clone F1) in immunofluorescence assay (sample n= 30; background n= 5). Error bars display standard error of the mean.

(D) Phase-contrast microscopic images showing the morphology of *SALL4*-WT and *SALL4*-het-KO (clone F1) iPSC colonies at 4x (top) and 10x (bottom).

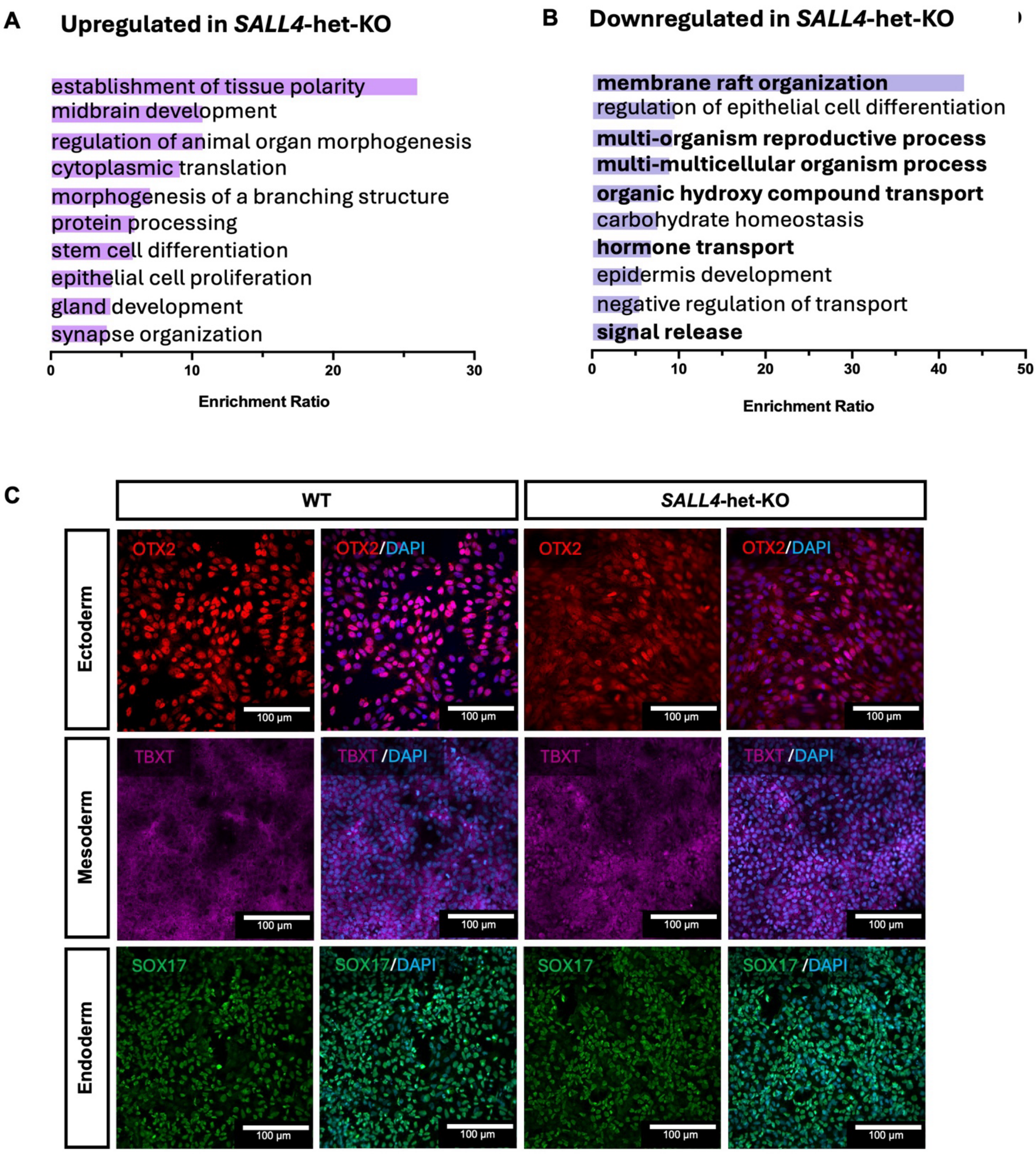

Fig. S3.Loss of *SALL4* does not impact ability of iPSCs to differentiate into the three germ layers cells

(A) Top 10 GO term enrichment for upregulated genes in *SALL4*-het-KO iPSCs determined using WebGestalt[71] over-representation pathway analysis, using affinity propagation as parameter for redundancy removal. All terms are non-significant (FDR > 0.05).

(B) Top 10 GO term enrichment for downregulated genes in *SALL4*-het-KO iPSCs determined using WebGestalt[71] over-representation pathway analysis, using affinity propagation as parameter for redundancy removal. In bold terms with FDR < 0.05.

(C) Immunofluorescence for ectodermal (OTX2), mesodermal (TBXT), and endodermal (SOX17) markers in *SALL4*-WT and *SALL4*-het-KO (clone F1) after trilineage differentiation. Scale bar: 100 µm.

Development • Supplementary information

A. GATING STRATEGY FIG. 3B

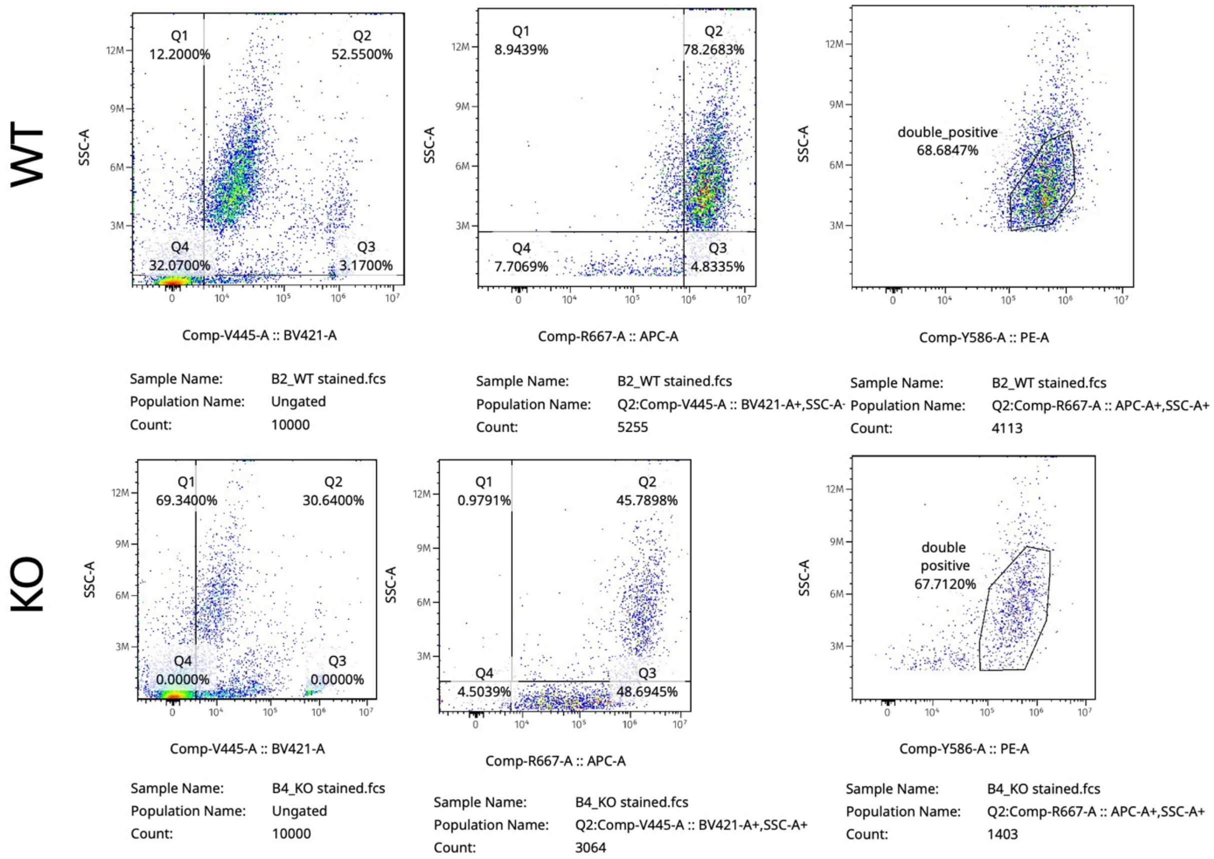

B. GATING STRATEGY FIG. 7B

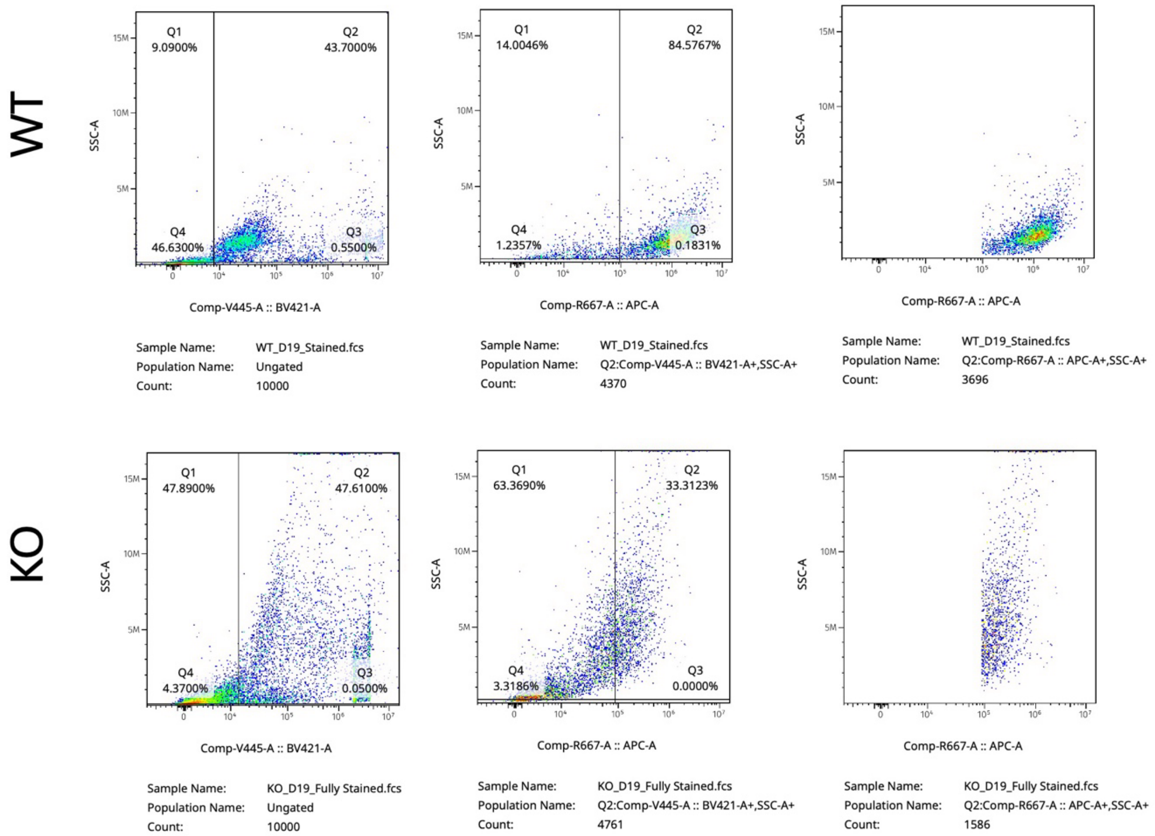

Fig. S4. Gating strategies for flow cytometry analyses. As reported in (A) figure 3B and (B) figure 7B.

Development • Supplementary information

A

SALL4 CUT&RUN Day-0 specific motifs			
**MOTIF #**	**Motif Consensus**		**p-value**
51	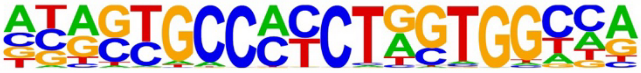	CTCF BORIS	$1e^{-270}$
260	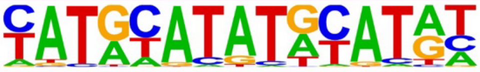	OCT:OCT	$1e^{-106}$

B

Day-0 specific SALL4 binding sites – *de novo* motifs				
**Motif Consensus**	**% of Target**	**% of Background**	**p-value**	**Closest match**
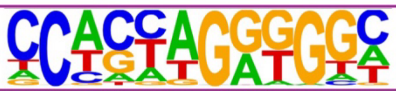	20.17%	2.23%	$1e^{-486}$	CTCF BORIS
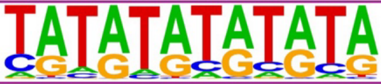	4.63%	0.40%	$1e^{-118}$	
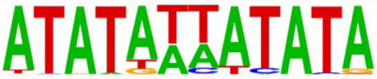	2.60%	0.17%	$1e^{-73}$	SALL4 (ZFC4) ARID/HMG

C

Day-5 specific SALL4 binding sites – *de novo* motifs				
**Motif Consensus**	**% of Target**	**% of Background**	**p-value**	**Closest match**
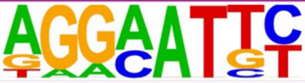	51.16%	17.48%	$1e^{-1613}$	TEAD
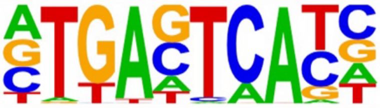	34.74%	10.61%	$1e^{-1108}$	AP-1
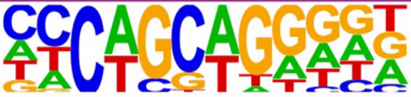	26.53%	9.00%	$1e^{-698}$	NANOG

Fig. S5. SALL4 does not bind to at-rich regions when relocating to CNCC-associated enhancers

(A) Table of motifs enriched in Day-0 specific SALL4-bound regions as determined using HOMER[68]. (B) Table of de novo motifs enriched in Day-0 specific SALL4-bound regions as determined using HOMER[68]. (C) Table of de novo motifs enriched in Day-5 specific SALL4-bound regions as determined using HOMER[68].

Development • Supplementary information

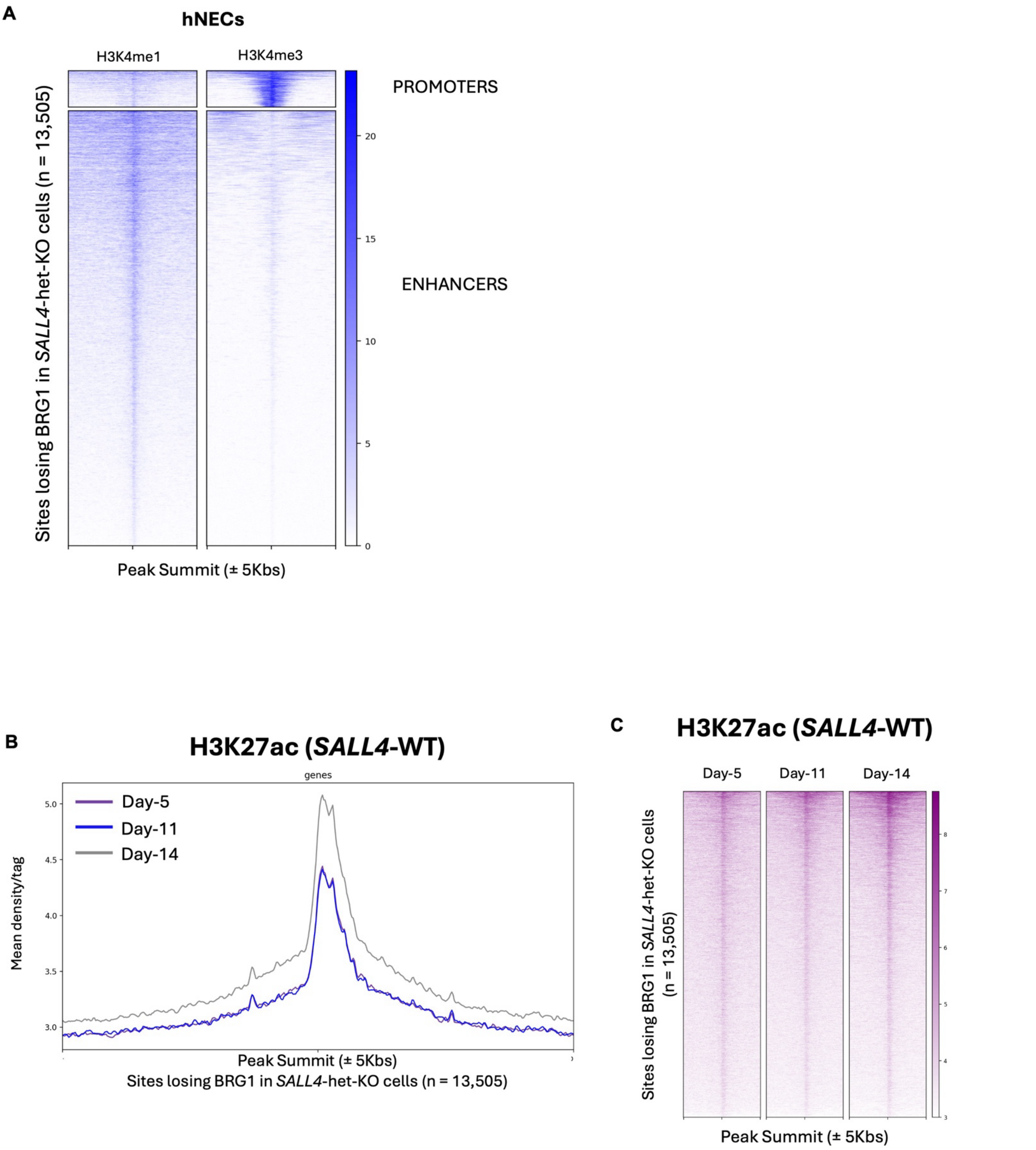

Fig. S6. CNCC-associated enhancers bound by SALL4 gradually gain H3k27ac mark

(A)ChIP-seqsignalintensityforH3K4me1andH3K4me3plottedacross±5kbofsiteslosingBRG1in SALL4-het-KO cells regions (n= 13,505) in human neuroectoderm-like cells (hNECs), centered on peak summits. Rows represent individual peaks ranked by signal intensity.

(B) Average profiles and (C) heatmaps showing H3K27ac ChIP-seq signal genomic sites plotted across ±5 kb of sites losing BRG1 in *SALL4*-het-KO cells regions (n= 13,505) at Day-5, Day-11, and Day-14 of the iPSC-to-CNCC specification. Centered on peak summits.

Development • Supplementary information

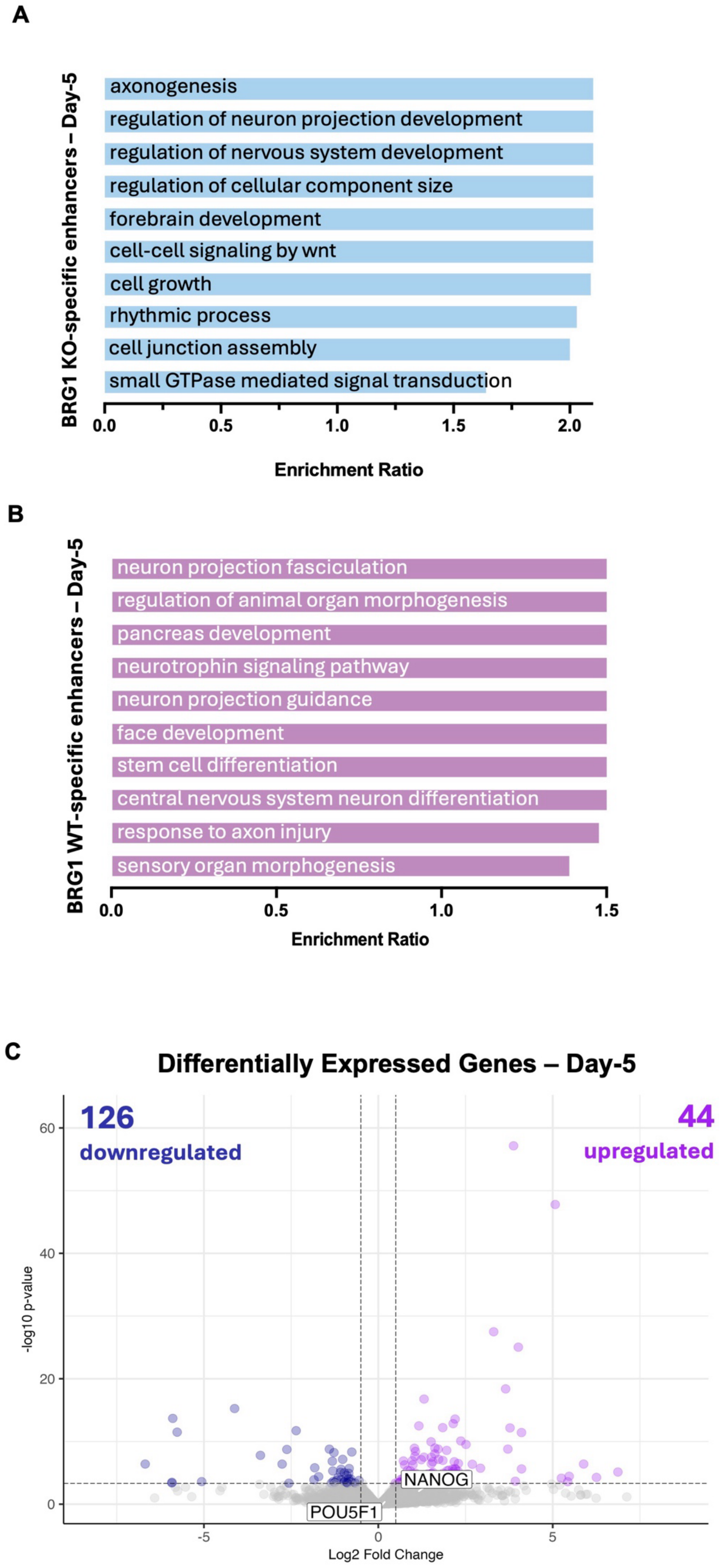

Development • Supplementary information

Fig. S7. BRG1 is relocated to neurogenesis-associated regulatory regions in absence of SALL4

(A) GO term enrichment for genes near BRG1 ChIP-seq peaks gained in *SALL4*-het-KO cells. GO terms were determined using WebGestalt[1] over-representation pathway analysis, using affinity propagation as parameter for redundancy removal (FDR<0.05).

(B) GO term enrichment for genes near BRG1 ChIP-seq peaks lost in *SALL4*-het-KO cells. GO terms were determined using WebGestalt[1] over-representation pathway analysis, using affinity propagation as parameter for redundancy removal (FDR<0.05).

(C) Volcano plot of genes differentially expressed in *SALL4*-het-KO relative to *SALL4*-WT in neuroectoderm-like cells (Day-5). Blue dots (n= 126) represent downregulated genes with p-adj < 0.05 and log2FoldChange < -1.5. Purple dots (n= 44) represent upregulated genes with p-adj < 0.05 and log2FoldChange > 1.5.

Development • Supplementary information

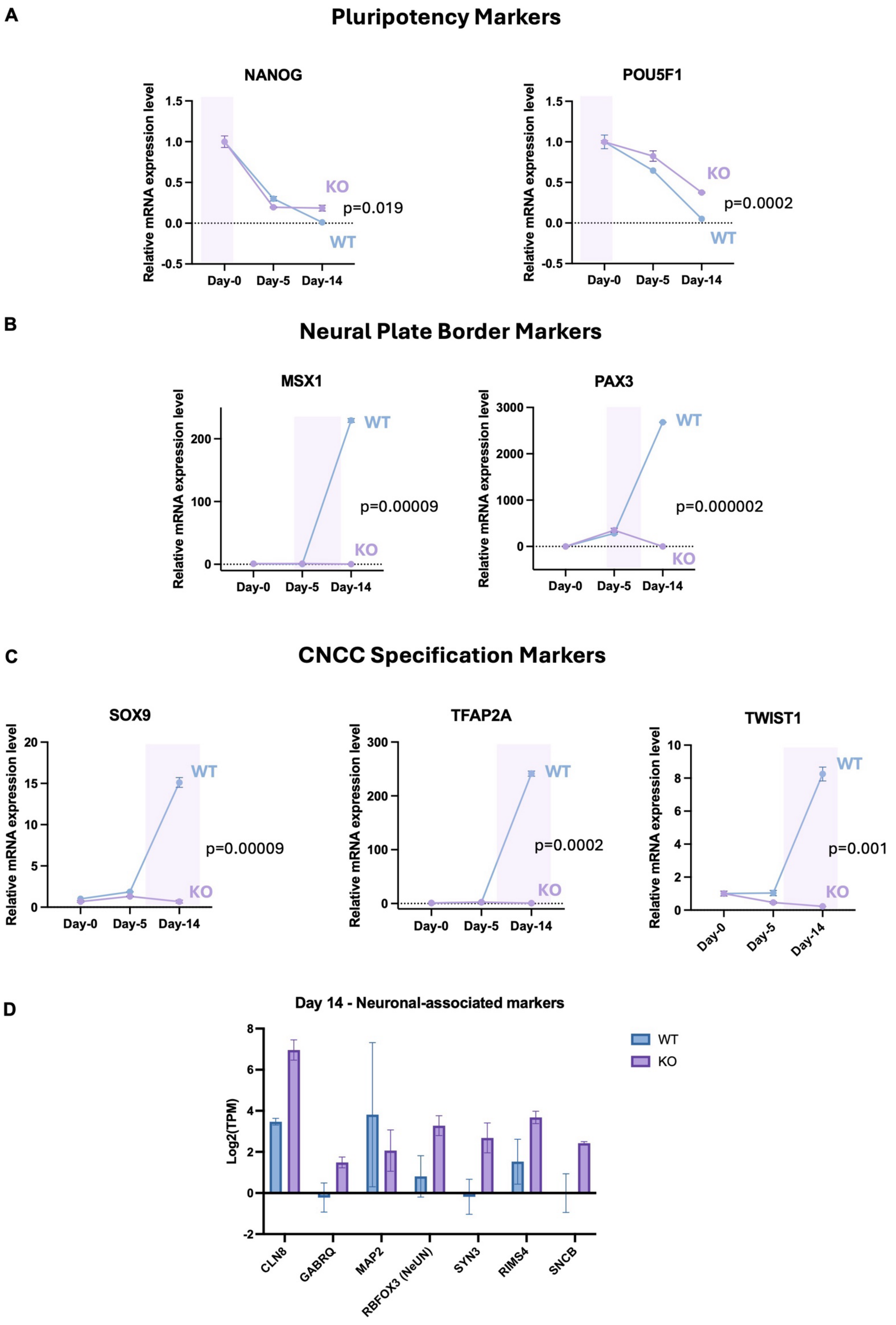

Development • Supplementary information

Fig. S8. CNCC but not NPB and pluripotency markers are impacted by absence of SALL4

Trajectory of stage-specific markers at Day-0 (n=2), Day-5 (n=2), and Day-14 (n=2). Displayed are the relative mRNA expression for:

(A) pluripotency markers (POU5F1 and NANOG),

(B) neural plate border markers (MSX1, PAX3),

(C) CNCC specification markers (SOX9, TFAP2A).

Highlighted are the approximate stages in which each marker is conventionally expressed in *SALL4*-WT.

(D) Expression (Log2[TPMs]) of neuronal-associated markers at Day-14.

Development • Supplementary information

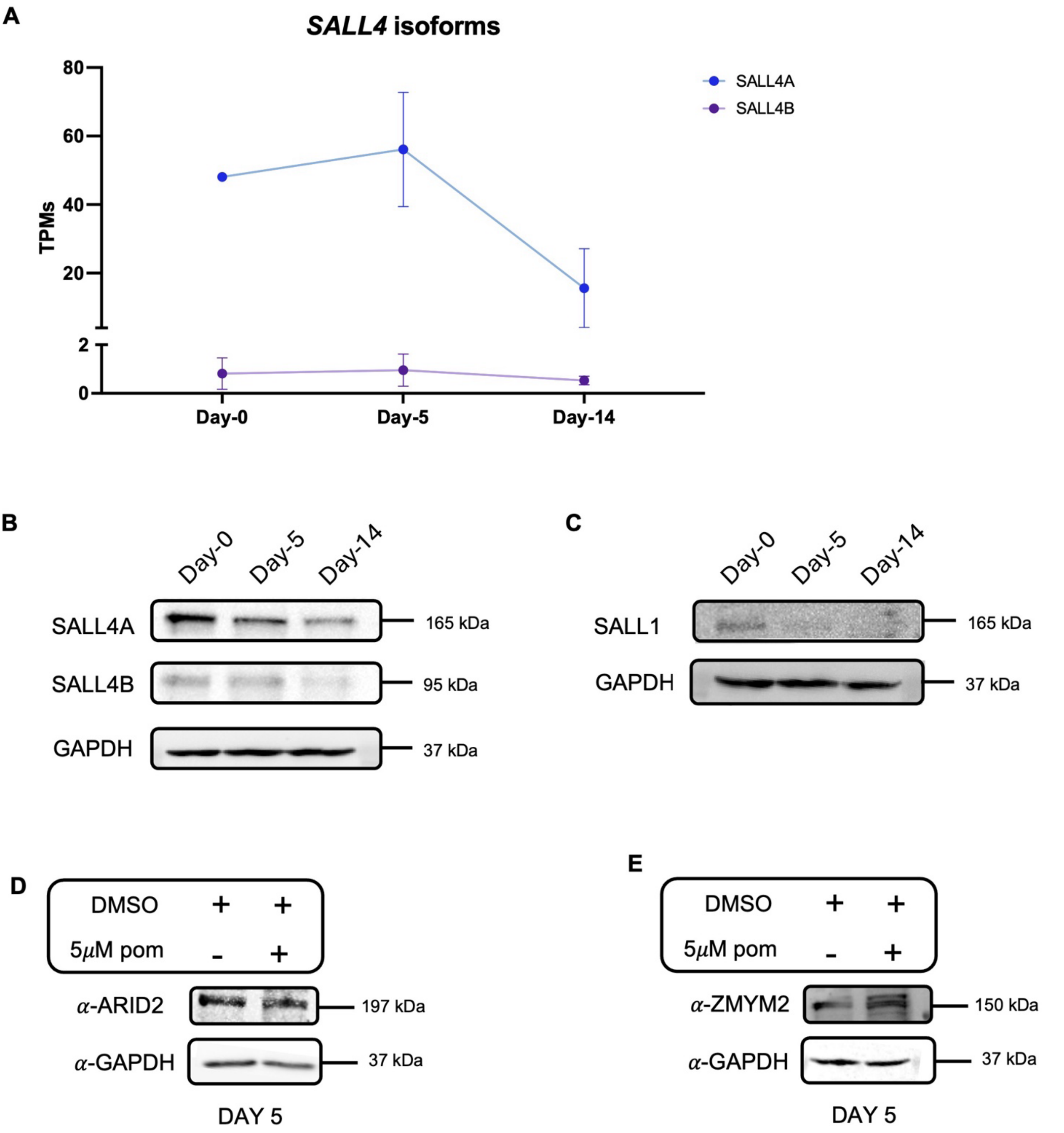

Fig. S9. Other CNCC-associated imids neosubstrates are not degraded after pomalidomide treatment

(A) Trajectory of SALL4A and SALL4B RNA expression (TPMs) at significant iPSC-to-CNCC specification time-points: Day-0 (n=2); Day-5 (n=3); Day-14 (n=2).

(B) Time-course immunoblot for SALL4 in SALL4-WT at Day-0, Day-5, and Day-14. GAPDH was used as a loading control.

(C) Time-course immunoblot for SALL4 in SALL4-WT at Day-0, Day-5, and Day-14. GAPDH was used as a loading control.

(D) Immunoblot for ARID2 in SALL4-WT at Day-5 after 24-hour treatment with DMSO (control) or 5µM pomalidomide (5µM POM). GAPDH was used as a loading control.

(E) Immunoblot for ZMYM2 in SALL4-WT at Day-5 after 24-hour treatment with DMSO (control) or 5µM pomalidomide (5µM POM). GAPDH was used as a loading control.

Development • Supplementary information

Table S1. List of AlphaFold predicted contacts between SALL4A and DPF2.

Available for download at
https://journals.biologists.com/dev/article-lookup/doi/10.1242/dev.205248#supplementary-data

Table S2. List of AlphaFold predicted contacts between SALL4B and DPF2.

Available for download at
https://journals.biologists.com/dev/article-lookup/doi/10.1242/dev.205248#supplementary-data

Table S3. List of TPMs and DEGs (RNA-seq) for Day-0, Day-5, and Day14.

Available for download at
https://journals.biologists.com/dev/article-lookup/doi/10.1242/dev.205248#supplementary-data

Table S4. List of nearest genes to SALL4 CUT&RUN peaks and BRG1 ChIP-seq peaks.

Available for download at
https://journals.biologists.com/dev/article-lookup/doi/10.1242/dev.205248#supplementary-data

Table S5. Reagents and resources

	SOURCE	IDENTIFIER	DILUTION
**Antibodies**			
Brg1 (D1Q7F)	Cell Signaling Technology	AB_2728743	5μg/mL
CD99 Antibody, anti-human, Vio® Bright B515, REAfinity™	Miltenyi Biotec	AB_2784331	2μg/mL
GAPDH (D16H11) XP Rabbit mAb	Cell Signaling Technology	AB_10622025	1:1000
Human Nanog Antibody	R&D Systems	AB_355097	1:500
Human/Mouse Oct-3/4 Antibody	R&D Systems	AB_354975	1:500
SALL1 Polyclonal Antibody	Thermo Fisher Scientific	AB_2900498	1:1000
Sall4 (EE-30)	Santa Cruz Biotechnology	AB_1129262	1:2000
Sall4 (D16H12) Rabbit mAb	Cell Signaling Technology	AB_10949321	1:100
APC anti-human SSEA-4	Biolegend	AB_2616818	4μg/mL
PE anti-human TRA-1-60-R	Biolegend	AB_1279447	4μg/mL
Rabbit IgG, monoclonal [EPR25A] Antibody	Abcam	AB_2687931	1:100
AP2 alpha Monoclonal Antibody (3B5)	Thermo Fisher Scientific	AB_2199412	1:500
CD326 (EpCAM) Monoclonal Antibody (1B7), eBioscience	Thermo Fisher Scientific	AB_795876	1:500
Anti-SOX9 antibody	Abcam	AB_2715497	1:500
Vimentin Polyclonal Antibody	Thermo Fisher Scientific	AB_2216267	1:500
PAX6 antibody	Abcam	AB_305110	1:500
ARID1B antibody	Abcam	AB_2243092	6μg/mL
ARID2 Polyclonal Antibody	Bethyl Laboratories	AB_1731041	1:1,000
ZMYM2 Polyclonal Antibody	Thermo Fisher Scientific	AB_11152210	1:1,000
IRDye 800CW Goat anti-Rabbit IgG	LI-COR Biosciences	AB_621843	1:15,000
Goat polyclonal Secondary Antibody to Mouse (IRDye® 800CW)	LI-COR Biosciences	AB_10859231	1:15,000
Donkey anti-Rabbit IgG (H+L) Highly Cross-Adsorbed Secondary Antibody, Alexa Fluor™	Thermo Fisher Scientific	AB_2762833	1:500

Development • Supplementary information

Plus 488			
Donkey anti-Mouse IgG (H+L) Highly Cross-Adsorbed Secondary Antibody, Alexa Fluor™ Plus 647	Thermo Fisher Scientific	AB_2762830	1:500
Goat anti-Chicken IgY (H+L) Secondary Antibody, Alexa Fluor™ 488	Thermo Fisher Scientific	AB_2534096	1:500

	SOURCE	IDENTIFIER
**Critical commercial assays**		
ATAC-Seq Kit	Active Motif	53150
Human Pluripotent Stem Cell Functional Identification Kit	R&D Systems	SC027B
Monarch® Total RNA Miniprep Kit	New England Biolabs	T2010S
Maxima First Strand cDNA Synthesis Kit for RT-qPCR	Thermo Fisher	K1641
NEBNext® Ultra™ II Directional RNA Library Prep Kit for Illumina®	New England Biolabs	E7760L
CUT&RUN Assay Kit	Cell Signaling Technology	86652
NEBNext® Ultra™ II DNA Library Prep Kit for Illumina®	Cell Signaling Technology	E7645L
Experimental models: Cell lines		
PENN123i-SV20	iPSC Core of the University of Pennsylvania	CVCL_EL23
PENN123i-SV20 *SALL4*-het-KO iPSC	Synthego	

	SOURCE	IDENTIFIER
**Oligonucleotides**		
18S	5′-ATACATGCCGACGGGCGCTG-3′	5′-AGGGCTGACCGGGTTGGTT-3′
TFAP2A	5′-GCCTCTCGCTCCTCAGCTCC-3′	5′-CGTTGGCAGCTTTACGTCTCCC-3′
NR2F1	5′-ATCGTGCTGTTCACGTCAGA-3′	5′-GCTCCTCACGTACTCCTCCA-3′
SOX9	5′-GTACCCGCACTTGCA	5′-TCTCGCTCTCGTTCAG

Development • Supplementary information

	CAAC-3′	AAGTC-3′
NANOG	5′-ATGCCTCACACGGAGACTGT-3′	5′-AAGTGGGTTGTTTGCCTTTG-3′
POU5F1	5′-TCGAGAACCGAGTGAGAGG-3′	5′-GAACCACACTCGGACCACA-3′
MSX1	5′-GAAGATGCGCTCGTCAAAGC-3′	5′-CTTACGGTTCGTCTTGTGTTTG-3′
PAX3	5′-ACAACGCCTGACGTGGAG-3′	5′-ATTTACTTCTCAGGATGCGGCT-3′
TWIST1	5′-GCCAGGTACATCGACTTCCTCT-3′	5′-TCCATCCTCCAGACCGAGAAGG-3′

	SOURCE	IDENTIFIER
**Software and algorithms**		
AlphaFold3	https://alphafoldserver.com/	SCR_025454
TrimGalore!	https://www.bioinformatics.babraham.ac.uk/projects/trim_galore/	SCR_011847
FlowJo Software version 10.9		SCR_008520
BEDTools		SCR_006646
Deeptools		SCR_016366
R Studio version 4.4.3		
GraphPad Prism 10.2.3	http://www.graphpad.com/faq/viewfaq.cfm?faq=1362	SCR_002798
DESeq2		SCR_015687

Development • Supplementary information
